# Investigating Visual Crowding of Objects in Complex Real-World Scenes

**DOI:** 10.1177/2041669521994150

**Published:** 2021-04-28

**Authors:** Ryan V. Ringer, Allison M. Coy, Adam M. Larson, Lester C. Loschky

**Affiliations:** Department of Psychology, Wichita State University, Wichita, Kansas, United States; Department of Psychological Sciences, Kansas State University, Manhattan, Kansas, United States; Department of Psychology, University of Findlay, Findlay, Ohio, United States; Department of Psychological Sciences, Kansas State University, Manhattan, Kansas, United States

**Keywords:** crowding, object recognition, scene perception, spatial selection/modulation, peripheral vision

## Abstract

Visual crowding, the impairment of object recognition in peripheral vision due to flanking objects, has generally been studied using simple stimuli on blank backgrounds. While crowding is widely assumed to occur in natural scenes, it has not been shown rigorously yet. Given that scene contexts can facilitate object recognition, crowding effects may be dampened in real-world scenes. Therefore, this study investigated crowding using objects in computer-generated real-world scenes. In two experiments, target objects were presented with four flanker objects placed uniformly around the target. Previous research indicates that crowding occurs when the distance between the target and flanker is approximately less than half the retinal eccentricity of the target. In each image, the spacing between the target and flanker objects was varied considerably above or below the standard (0.5) threshold to either suppress or facilitate the crowding effect. Experiment 1 cued the target location and then briefly flashed the scene image before participants could move their eyes. Participants then selected the target object’s category from a 15-alternative forced choice response set (including all objects shown in the scene). Experiment 2 used eye tracking to ensure participants were centrally fixating at the beginning of each trial and showed the image for the duration of the participant’s fixation. Both experiments found object recognition accuracy decreased with smaller spacing between targets and flanker objects. Thus, this study rigorously shows crowding of objects in semantically consistent real-world scenes.

## Visual Crowding in Real-World Scene Perception

We see the scenes surrounding us in our visual world with both central and peripheral vision. Our entire horizontal visual field extends to roughly 210° in diameter, however, much of our conscious experience occurs within a 5° to 8.5° radius central window (i.e., central vision), which contains the macula and the highest density of photoreceptors (for review, see Strasburger, 2020). Therefore, most of our visual environment is processed using peripheral vision. This raises a key question that vision scientists have been investigating for more than the last 100 years—what is the nature of vision in our visual periphery (for review, see Loschky et al., 2017; Strasburger et al., 2011; Wilson et al., 1990)? Here we are concerned with a particular visual phenomenon known as *crowding*, which limits both our conscious perception of things in peripheral vision and our ability to recognize them. Crowding has been studied relatively intensively over the last two decades, including special issues of the *Journal of Vision* in both 2007 (Pelli, Cavanagh, et al., 2007) and in 2014 to 2017 (Crowding: New Vistas,2014–2017), and several in-depth review articles (Levi, 2008; Pelli & Tillman, 2008; Strasburger, 2020; Strasburger et al., 2011; Whitney & Levi, 2011), but an important and unanswered question is, does it operate for objects in real-world scenes? If crowding can affect object recognition in real-world scenes, then it may place very important constraints on our experience of the visual world.

Visual crowding has been characterized as impaired object or form recognition due to the presence of surrounding objects (Bouma, 1970; Ehlers, 1936; Stuart & Burian, 1962; Woodrow, 1938). The most fundamental aspect of crowding is the existence of a critical spacing, which is defined by the separation between the centers of the target and the flanking object that is necessary to achieve recognition performance equivalent to that of a target presented in isolation. It was discovered that the critical spacing between target and distractor objects for a given eccentricity was equal to approximately ½ the visual angle of the retinal eccentricity of the target object (Bouma, 1970), which has come to be known as Bouma’s constant (Whitney & Levi, 2011) or Bouma factor (Pelli et al., 2004; Pelli, Tillman, et al., 2007; Strasburger, 2020). It has been replicated across a number of studies and a range of stimuli (Latham & Whitaker, 1996; Pelli et al., 2004; Pelli & Tillman, 2008; Whitney & Levi, 2011). Whether nearby objects are separated by a distance either greater than or equal to the Bouma factor, or less than the Bouma factor, determines, to a first approximation, whether they do or do not suffer impaired object discrimination, respectively.^1^

Although crowding and its properties have been extensively studied, previous studies of crowding may have only limited generalizability to seeing real-world objects in scenes in peripheral vision. The majority of crowding studies have used simple stimuli such as alphanumeric characters (Strasburger et al., 1991), Gabor patches (Parkes et al., 2001; Poder & Wagemans, 2007), or Vernier acuity stimuli (Levi et al., 1985). Nevertheless, studies utilizing complex displays of these simple stimuli have found important contextual effects of flanking objects on targets, where targets and flankers are more likely to be pooled together when they conform with Gestalt grouping principles (for reviews, see Herzog & Manassi, 2015; Herzog et al., 2015). However, the familiar structure of our environment has facilitated more efficient extraction of information in the environment, and thus high-level scene representations may provide more functional separation between targets and flankers, especially if the Gestalt of the flanking objects extends away from the target object (Van der Burg et al., 2017).

Only recently has crowding of real-world stimuli begun to be investigated. One study investigated crowding of realistic objects on a blank gray background (e.g., a rubber ducky crowded on the vertical meridian by a lamp and watering can) and produced results similar to those from experiments using simpler stimuli (Wallace & Tjan, 2011). While that study was an important advance in the direction of investigating crowding of ecologically valid stimuli, it did not situate the objects in realistic scene contexts, as they would occur in the real world. A step in this direction was recently taken by creating an image to demonstrate crowding of one real-world object, a boy, in one real-world scene, a street (Whitney & Levi, 2011). While the image is a compelling demonstration, it also highlights the lack of empirical studies to both quantify and test the generality of the phenomenal experience engendered by the demonstration.^2^ Another study by Wallis and Bex (2012) investigated crowding in natural scenes using so-called *dead leaves*, which were synthetic texture patches embedded in various locations and sizes within the image. The results from this study show that, while participants’ ability to detect the leaves varied as a function of patch size and eccentricity, detection was impaired when the *dead leaves* appeared in regions of the scene where structural complexity was high. While this does not demonstrate crowding of real-world objects in natural scenes, it does demonstrate how real-world environments can contribute to crowding.

A more recent study investigated the detection of vulnerable road users (VRUs) such as pedestrians, motorcyclists, and bicyclists in traffic scenes (Sanocki et al., 2015). The authors found that VRU detection was significantly impaired when vehicles flanked the VRUs, with the presence or absence of flanking vehicles defining the crowding manipulation (Sanocki et al., 2015). Interestingly, the crowding manipulation showed participants’ bias becoming more conservative in crowded scenes, relative to uncrowded scenes. The fact that flanking objects decreased overall object detection could be argued to be inconsistent with previous results showing that crowding does not affect *detection* of objects, only their identification. Specifically, one could argue that the results of Sanocki et al. either suggest that crowding operates differently in real-world scenes than with simpler stimuli, or that the phenomenon was something other than crowding. The latter possibility cannot be dismissed easily because Sanocki et al. did not explicitly account for the retinal eccentricity of targets and flanking objects, nor did they calculate the target–flanker spacing to interpret their resulting data. However, an alternative argument is that the “VRU detection task” did, in fact, involve identification (or superordinate level categorization), rather than detection, and that their results are therefore consistent with prior crowding results. Specifically, the target VRUs came from a variety of different basic level categories (i.e., pedestrians, bicyclists, and motorcyclists) and had to be identified as members of the superordinate VRU category. Finally, Sanocki et al. leave open the question of whether their effects on detection of VRUs actually involved crowding, per se, rather than some related phenomenon (e.g., masking, attentional capture).

Given that crowding has been argued to be ubiquitous in spatial vision (Levi, 2008), an alternative question could be, is there any good reason *not* to expect crowding of objects in real-world scenes? In fact, there are. Specifically, if crowding of objects occurred ubiquitously in natural scenes, one might assume that peripheral object recognition in scenes should be difficult, if not impossible. Yet, there is evidence that people are able to recognize objects (animals) in natural scenes presented in their visual periphery, even as far as 70° eccentricity (Thorpe et al., 2001).

One study which has rigorously investigated whether crowding can occur in natural scenes did so by comparing peripheral object recognition for objects completely isolated from their scenes, versus the same objects viewed through apertures (“windows”) that showed increasingly more surrounding context (Wijntjes & Rosenholtz, 2018). If crowding were occurring, the isolated objects would be easiest to recognize. Furthermore, as more surrounding context was revealed, performance would become increasingly degraded. However, the results showed exactly the opposite pattern, with the poorest recognition performance occurring when objects were isolated, and increasingly better recognition performance occurring as more surrounding context was revealed (Wijntjes & Rosenholtz, 2018). The authors argued that any detrimental effects of crowding produced by flanking objects in scenes was more than compensated for by the benefits of context (e.g., by priming scene-consistent object representations). This is consistent with the idea that the gist of a scene facilitates recognition of objects in it (Biederman et al., 1982; Boyce et al., 1989; Davenport & Potter, 2004). Thus, there is good reason to expect little, if any, crowding effect for objects viewed peripherally in scenes. However, there is an equally compelling alternative explanation of their results. It is important to note that the authors produced no positive evidence of crowding in any condition—they only showed negative evidence. Thus, Occam’s razor suggests that the simplest explanation of their results is an issue of construct validity, and that there was no crowding to begin with. If so, how can they explain the lack of crowding in their natural scenes, when we expect it to be there? Here, it is important to note that, as with the study by Sanocki et al. (2015), Wijntjes and Rosenholtz (2018) did not manipulate target–flanker spacing when attempting to evoke the conditions for crowding. Instead, in their first experiment, they varied the size of an aperture which allowed for the *possibility* of nearby objects to flank the target as they were revealed in the scene image (Wijntjes & Rosenholtz, 2018). Thus, it is plausible that the surrounding scene contexts were simply not cluttered enough to produce clear evidence of crowding. If so, sufficient clutter might have produced clear evidence of crowding in natural scenes. In Experiment 2 of Wijntjes and Rosenholtz (2018), participants categorized objects which were either isolated or occluded from the scene. Surprisingly, both conditions yielded approximately 40% accuracy (with chance being 1.2% correct), suggesting that the scene background itself produced object predictions that were roughly equal to the ability to recognize objects in peripheral vision in the absence of scene context. This suggests that, during the object recognition process, the presence a scene context may reduce the potential number of candidate objects that *could* be in peripheral vision (Wijntjes & Rosenholtz, 2018). Nevertheless, the question remains whether crowding can occur in natural scenes at all. Without varying the target/flanker relationship while holding scene/object context constant, *whether* and *when* we should expect to find crowding of objects in natural scenes remains to be determined.

Thus, this study addresses a key question for crowding research, namely, whether there is compelling evidence of crowding of real-world objects in real-world scenes as has been shown for simpler stimuli (e.g., letters, numbers, or Gabor patches) on blank screens, or is the crowding effect overcome by the benefit of having objects in the context of semantically consistent scenes? As noted earlier, there is scant empirical evidence of crowding of realistic objects in realistic real-world scenes along with evidence *against* the existence of crowding in scenes, which may be due to facilitation of object recognition by scene context. Therefore, the chief goal of this study was to rigorously experimentally test for a crowding effect on recognition of real-world objects in real-world scenes. If facilitation of object recognition by consistent scene context is sufficient to overcome any crowding effects, then we should find little if any evidence of crowding in scenes containing objects consistent with their scene backgrounds. We therefore produced scenes containing only scene-consistent objects. However, to rigorously test for the existence of crowding in scenes, we have manipulated both the retinal eccentricities of target objects and the spacing between the centers of the target and flanking distractor objects. We have then compared spacing conditions that would be predicted to produce more crowding versus less. We did this by using architectural rendering software to produce highly realistic scenes in which we could precisely control the locations and spacing of target and distractor objects. Furthermore, because the most compelling demonstration to date of crowding in a realistic scene included only a single target object and scene, in order to produce more generalizable results, we produced several scenes with multiple objects in multiple locations.

## Experiment 1: Tachistoscopic Presentation

### Method

#### Participants

Forty-four undergraduates from Kansas State University (21 females) gave informed consent to participate in the experiment for course credit. Participants had a mean age of 20.0 years and all had visual acuity of 20/30 or better.

#### Stimuli

**Figure 1.**
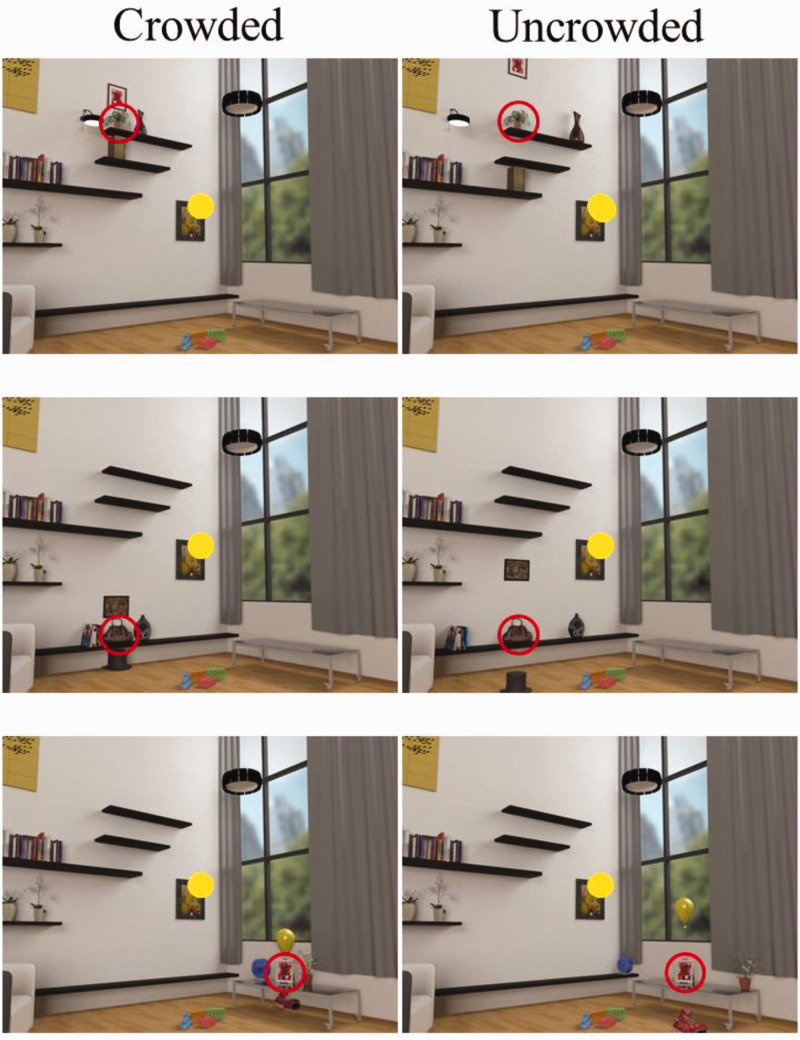
Sample scenes showing examples of crowded (left) and uncrowded (right) targets within a single base-image. The yellow circle in the center of each scene represents the central fixation point. The object located within the red ring is the target object to be identified. Note that the red circles shown in this figure only indicate the target for demonstrative purposes and were not included in the actual experiment. Within each base-scene, six versions were generated: three versions with different target objects and locations, and a crowded and uncrowded version for each target object. Participants saw each unique target once.

**Figure 2.**
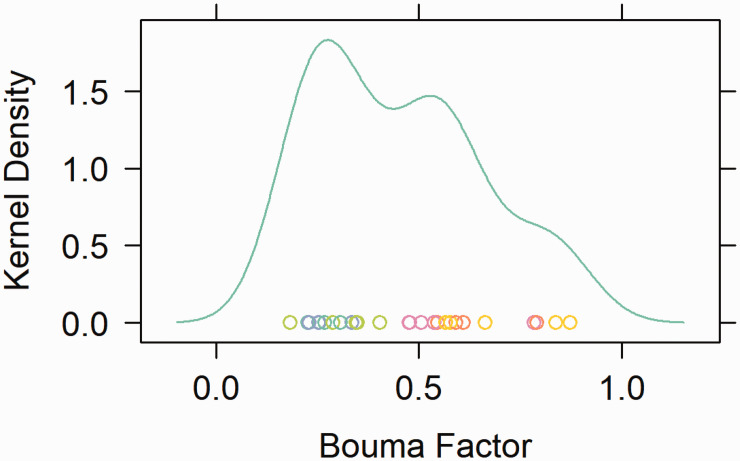
The distribution of flanker/target spacing ratio (the Bouma factor) values or the ratio of the flanker-to-target spacing and the retinal eccentricity of the target. Each point represents a single image version of a given base image, and its color represents its base image. *X*-values on the plot represent the Bouma factor, and *Y*-values represent their frequency, which has been smoothed along the *x*-axis using the kernel density function of the *lattice* library in R.

Computer generated living room scenes were created using Autodesk 3ds Max® design software.^3^ Eight different base images were created, with six versions of each base image: three crowded and three uncrowded (see Figure 1). The objects and backgrounds used were available within the software library. All scene images were 1,024 × 768 pixels and 37 × 27.5 cm. At the viewing distance of 53.3 cm, images subtended 38° × 29° of visual angle. Target objects were presented approximately 11.3° to 13.8° (*M* = 12.0°, standard deviation [*SD*] = 1.0°) from the center of fixation at eight potential locations. Four flanker objects were placed around the target object, with the distance between the target and flankers varying in a way that would make the crowding effect more or less likely to occur. The spacing between flankers and target objects ranged between 2.5° and 9.8° (*M* = 5.75°, *SD* = 2.39°), and the *Bouma factor*,^4^ the ratio between the mean visual angle between the target and flankers and target retinal eccentricity, ranged between 0.18 and 0.87 (*M* = .45, *SD* = 0.20; see Figure 2). Note that crowding typically diminishes when the Bouma factor is above a critical value of 0.4 (Pelli et al., 2004; Pelli, Tillman, et al., 2007; Strasburger, 2020; Strasburger et al., 1991). The stimuli were constructed so that both the overall scene configuration and the arrangement of target and distractor objects appeared natural and realistic for both the crowded and uncrowded conditions. Objects were placed in scene-consistent locations and all items that appeared within the scene could reasonably be expected to appear in a living room.

#### Procedure

Participants’ visual acuity was tested using a Snellen eye chart, and all had 20/30 vision or better. Participants were then given instructions for the experimental procedures. Participants viewed images using a chin rest to maintain a constant viewing distance throughout the experiment and between participants. To familiarize participants with the names associated with each of the objects, participants went through a learning phase, in which each object was presented at the center of the screen on a neutral gray background for 750 milliseconds followed by its label, which remained on the screen until the participant clicked a mouse to proceed to the next object. A total of 50 objects were presented in the learning phase. Following the learning phase, participants completed six practice trials. Half of the trials included crowded objects and half uncrowded, and participants were given feedback after each trial, to allow them to become familiar with the task and its difficulty level.

#### Trial Procedures

**Figure 3.**
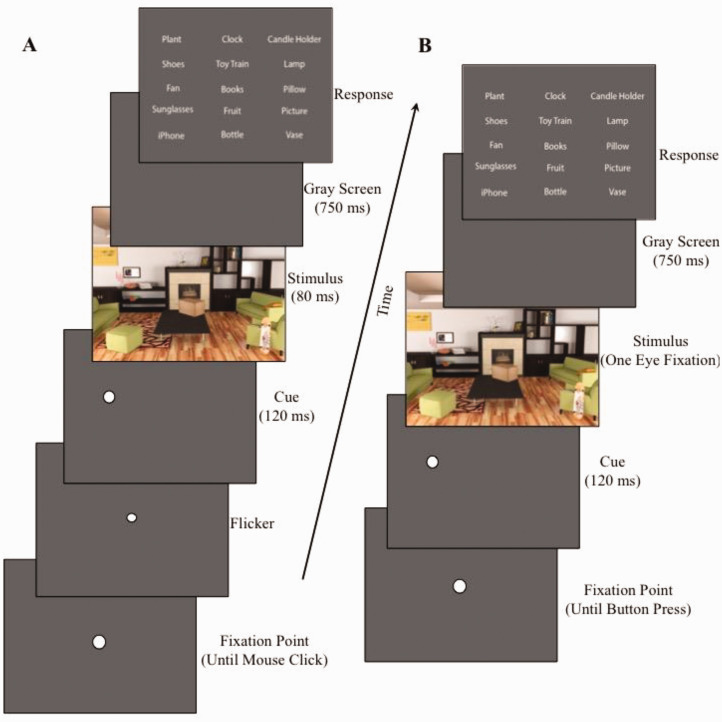
Trial schematics for Experiments 1 and 2. A: Schematic for Experiment 1, utilizing a tachistoscopic presentation. B: Schematic for Experiment 2, utilizing eye tracking and a gaze-contingent presentation.

At the beginning of the trial, the participant was presented with a fixation dot (see Figure 3A). To begin the trial, participants clicked the mouse button. A small white dot flickered 4 times at fixation (one flicker cycle: 36 milliseconds on, 24 milliseconds off) to capture attention at the center of the screen (Carmi & Itti, 2006; Ludwig et al., 2008; Mital et al., 2010). Next, a white dot, 0.5° in diameter, flickered at the target object’s location twice (one flicker cycle: 36 milliseconds on, 24 milliseconds off) as a sudden-onset cue to capture attention at the target location and help store information from the upcoming target in visual working memory (Belopolsky et al., 2008; Theeuwes et al., 1999). Then, a scene image containing the target object was presented for 80 milliseconds. Because the minimum normal saccadic latency is 150 milliseconds, and average saccadic durations are 50 milliseconds, if a participant quickly made a saccade to the cued target location, their eyes should have arrived there no earlier than 200 milliseconds after the cue onset, by which time the target would be extinguished. Then, the scene image was followed by a blank gray screen for 750 milliseconds. Participants responded by clicking on the target object’s category name from among a 15-alternative forced choice matrix containing the names of the target, its flankers, and all other objects used in that scene, but only on the other two trials for that scene. The arrangement of the object names on the response screen was randomized on each trial.

#### Design

Six different scenes were included in the experiment, with each scene containing three potential target objects. Each scene was shown only 3 times, once for each of three targets in that scene. However, to avoid having the participants learn the targets and distractors, only the specific target and its four specific distractors were shown on any given trial, and on only that single trial. The two other targets for that scene, and each of those targets’ specific distractors, were shown on the two other trials for that scene (see Figure 1). A total of 18 experimental trials were completed, with half of the trials displaying crowded targets and the other half uncrowded targets. No participant viewed both the crowded and uncrowded version of the same target. Scenes were presented in a random order, and the image-condition blocks (i.e., crowded vs. uncrowded) were counterbalanced across participants.

### Results

#### Precursors

With 44 participants each completing 18 trials, there were a total of 792 observations.^5^ Due to an error in stimulus creation and peripheral cue location in three base image pairs, 3 trials out of 18 were filtered out for each participant (i.e., 132 total observations were filtered). After the above filtering, analyses were carried out on a total of 660 observations.

#### Mean Object Identification Accuracy

In the study of crowding (and psychophysics in general) a two-stage analytical approach often used is to (a) identify each subject’s parameter estimates of their best-fitting psychometric function and (b) then subject these individual parameter estimates to a standard analysis of variance to determine whether they vary across predictors of interest. This analysis is typically performed on data collected over many trials using homogeneous stimuli, for instance, Gabor patch identification against a neutral-gray background. In contrast, this study estimated object identification performance in natural scenes, thus contributing an additional source of within-subject variance in the parameter estimates. Furthermore, not all participants saw the same version of each scene/object pairing, making it impossible to disentangle the influence of each image on each participant’s performance using the standard two-stage approach.

To address this issue, generalized linear mixed-effect modeling was used to calculate the (logistic) psychometric function (i.e., object recognition as a function of Bouma factor) while accounting for the independent sources of variability from subjects and stimuli. Mixed-effect models include fixed-effect predictors (e.g., the Bouma factor) while also modeling the random variation in each subject’s average accuracy, each subject’s sensitivity to within-subject variables (e.g., Bouma factor), and differences in the average difficulty of each scene. This random variation is not treated as the result of fixed effects but rather as the result of random sampling from a population of subjects and stimuli (Baayen et al., 2008). Because the analysis was performed at the individual trial level, the outcome variable (correct vs. incorrect response) was binomial, necessitating the use of a *generalized* linear model with a binomial distribution for the outcome variable and a logit link function (Jaeger, 2008) with the lme4 package (Bates et al., 2014) in the R statistical software (version 3.1.3).

Our criterion variable was object recognition accuracy (coded as 0 and 1 for incorrect and correct responses, respectively), therefore we specified a binomial distribution with a logit link function and used Bouma factor as our sole fixed effect (i.e., predictor; see Table 1, Column 1). With respect to random-effect structures, two classes of models were constructed to predict object recognition accuracy. The first class of models included by-subject random effects only, accounting only for individual participant differences (see Table 1, Column 2). The second class of models included both by-subject and by-item (base-image) random effects, which accounted for individual differences among participants as well as individual effects of each scene (see Table 1, Table 4). Within each of these two classes of models, three versions were generated. The first version included a random intercept with no fixed effects (i.e., a null model). The second version had a fixed effect of Bouma factor and a by-subject random intercept, which accounted for individual variability among participants’ mean accuracy, but did not account for individual differences when calculating the fixed effect (i.e., the slope) for Bouma factor. The third version had a fixed effect of Bouma factor, a by-subject random intercept and random slope for Bouma factor. This model version calculated the fixed effect (i.e., the slope) of Bouma factor on object recognition accuracy on the basis of individual subjects’ slopes. By-item random effects were included as random intercepts only, as not all participants viewed the same versions (i.e., crowded vs. uncrowded) of each image, and thus, these models accounted for the impact of the image when calculating the fixed effect of Bouma factor. Model fitness was evaluated through likelihood ratio tests between the models with the two highest log-likelihood ratio values (Bates et al., 2015).

**Figure 4.**
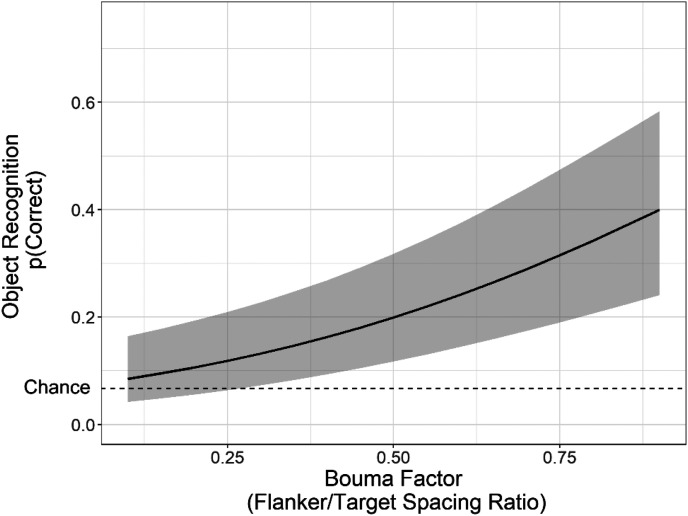
Fitted mean accuracy for object identification as a function of the fixed effect of Bouma factor, based on the optimally fit mixed-logit model from Experiment 1. The log-odds for object recognition have been transformed with an inverse-logit function to provide proportion correct for the logistic function. The range of Bouma factor extends from the minimum to the maximum values used in this study (as illustrated in Figure 2). Error bars represent a 95% confidence interval above and below the mean (using model parameter estimates obtained from lme4). The dotted line indicates the chance level of accuracy (6.7%).

The likelihood ratio tests demonstrated that the model which included Bouma factor as a fixed effect, and further included by-subject and by-item random intercepts, provided a significantly better fit than the null fixed-effect model with the same random-effect structure (−327.47 vs. −338.83, respectively; χ^2^ = 22.73, *df* = 1, *p* < .001).^6^ Although the model with by-subject random slopes and by-item random intercepts showed a slightly greater likelihood ratio value (−326.89), it was less parsimonious as it also had more free parameters and did not demonstrate significantly better fit (χ^2^ = 1.16, *df* = 2, *p* =.56). Therefore, the by-subject and by-item random-intercept model was selected. This model structure is given by the generalized linear equation (Equation 1):

(1) $$ln\frac{p}{1-p}=\alpha +\beta X+Z_{i}+Z_{j}$$

In this equation, the log-odds for target recognition accuracy (i.e., *ln* $\frac{p}{1-p}$ ) vary as a function of the intercept (α) and the fixed effect of Bouma factor, where *X* represents the centered.^7^ Bouma factor value for a given image and β represents the slope for Bouma factor. Additional model flexibility is provided by the random intercepts of Subject (*Z_i_*) and Base image (*Z_j_*). This model was selected to evaluate the fixed effect of Bouma factor on object recognition accuracy. Mean accuracy (i.e., the intercept) was low, approximately 17.9%, but well above the chance-level (6.7%) performance (α = −1.52, *z* = −4.73, *p* = .001). As shown in Figure 4, the slope for Bouma factor was significantly positive (β = 2.46, *z* = 4.76, *p* < .001), meaning that accuracy was lowest when the space between the target and flanker objects was small, but improved with increasing space. Thus, the crowding effect was found for objects in real-world scenes. However, the psychometric function presented in Figure 4 is clearly only a small portion of the overall function, with accuracy continuing to steepen at the upper limit of Bouma factors used in this study.

**Figure 5.**
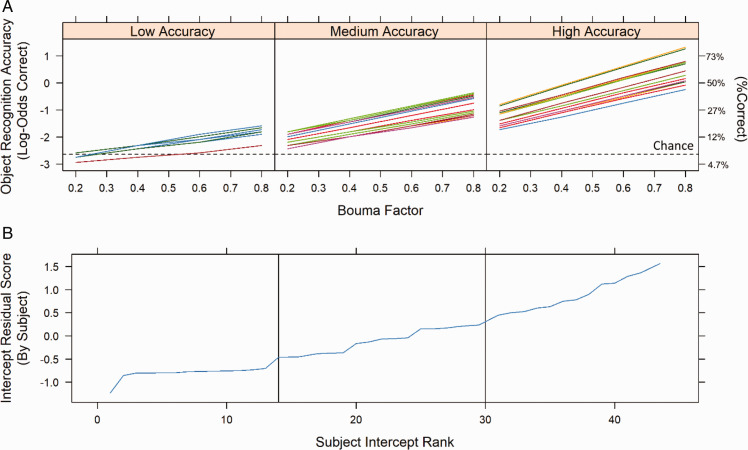
Fitted log-odds accuracy as a function of Bouma factor for individual participants in Experiment 1. A: Proportion correct is indicated on the right *y*-axis. Each line represents an individual participant’s fitted accuracy for the by-subject random slopes model (third row, far-right column of Table 1), and the panels are divided among participants with low (left), medium (center), or high (right) levels of mean accuracy for object recognition. Group divisions were based on random-effect residual scores in Figure 5B. The horizontal dotted line indicates chance-level performance. This is not the model used to generate Figure 4, because it has additional flexibility for individual slopes, whereas the optimally fit model for Experiment 1 only used random intercepts. Notice that only the subjects’ intercepts for object recognition accuracy vary between the three accuracy groups, even though slope is also left free to vary. B: Mean accuracy (intercept) residual scores for individual subjects. Individual subjects are ordered by mean accuracy rank, and the vertical lines indicate divisions used to generate the binned accuracy groups in Figure 5A.

In addition, the optimal model’s random effects included only a random intercept, indicating that there was substantial individual variability in terms of mean accuracy (i.e., the intercept) but not as a function of the effect of Bouma factor. This suggests that the effect of Bouma factor on recognition accuracy was quite uniform across participants (see Figure 5A). Furthermore, there was no systematic relationship between the fixed effects of intercept (i.e., individual subjects’ mean accuracy) and slope (i.e., individual subjects’ change in accuracy across Bouma factor), *r*(43) = −.10, *p* = .51.

**Table 1.** Model Fitness Comparisons for Experiment 1.

Fixed effects	By-subject random effects	Model log likelihood (by-subject only)	Additional by-item random effects	Model log likelihood (by-subject + by-item)
Null/intercept	Intercept	−352.47 (2)	Intercept	−338.83 (3) −327.47 (4)* −326.89 (6)
Bouma factor	Intercept	−343.66 (3)	Intercept	
Bouma factor	Bouma factor	−342.95 (5)	Intercept	

For more details on parameter estimates, see Equation 1 and Figure 4 in the main text.*Column 1:* The *Fixed Effects* structures for the models being compared. These include a null/intercept model on Row 1, which serves as a random chance/baseline comparison. Rows 2 and 3 include the fixed effect of Bouma factor.*Column 2:* The *By-Subject Random Effects* structures account for variability in object recognition across subjects. Rows 1 and 2 show random *Intercept* models, which account for individual differences for overall object recognition (i.e., random subject intercepts). Row 3, *Bouma factor*, includes models where the slope for Bouma factor can vary across participants (i.e., random subject slopes).*Column 3:* Log-likelihood values for the *By-Subject Only* random-effect models.*Column 4:* The *By-Item Random Effects* structures include measures of variability in object recognition across base images. Note that these models also include the By-Subject Random Effects from the same row of Column 2.*Column 5:* Log-likelihood values for the *By-Subject + By-Item* random-effect models.*Denotes the model with the combined largest log likelihood with the smallest *df* This model included the fixed effect of Bouma factor, the “By-Subject Random Effect” of Intercept, and the *By-Item Random Effect* of Intercept of base image. For more details on parameter estimates, see Equation 1 and Figure 4 in the main text.

### Discussion

This experiment provides a rigorous test of object crowding in real-world scenes. This was shown experimentally by comparing conditions predicted to produce crowding, with the Bouma factors ranging from 0.2 to over 0.8. It also used numerous (15) different realistic objects in several (6) different realistic scenes. The objects were also placed in scene-consistent locations within the scenes, which should enhance object identification (Boyce & Pollatsek, 1992; Davenport & Potter, 2004; Wijntjes & Rosenholtz, 2018, but see Hollingworth & Henderson, 1998). Likewise, the flankers at their respective spacings were also put in scene-consistent locations for the same reasons (see Figure 1). However, Experiment 1 had an important limitation. Specifically, all target presentations were presented at roughly equal distances from the center of the image. Thus, if participants noticed this, they could have adopted a strategy of quickly moving their eyes from the center of the screen in hope of fixating the target when it appeared, in which case they would not need to use their peripheral vision to accomplish the task. Wijntjes and Rosenholtz (2018) shared a similar concern in their study. To deal with that concern, in their study, the experimenter watched a live video image of participants in order to visually detect eye movements during each trial. We adopted a different approach to dealing with this concern, by carrying out a second experiment using an eyetracker to more carefully control for eye movements by participants.

## Experiment 2: Gaze-Contingent Presentation

To rule out the possibility of participants adopting a strategy of foveating the target object, which would invalidate the results of Experiment 1, we conducted a gaze-contingent version of the experiment using high spatial and temporal resolution eye tracking.

### Method

#### Participants

Seventy-three undergraduates from Kansas State University (53 females) gave informed consent to participate in the experiment for course credit. Participants had a mean age of 18.4 years and all had visual acuity of 20/30 or better.

#### Stimuli

The stimuli used were the same as those in Experiment 1.

#### Procedure

Procedures were exactly the same as in Experiment 1, with the following exceptions: Participants were given a demonstration of how their eye movements could affect the image shown on the screen through the use of a gaze-contingent display (see Figure 3B). This was intended to make participants more aware of their eye movements and help them maintain central fixation during the experiment, rather than trying to fixate the peripheral target in order to identify it. We used an EyeLink 1000 eyetracker with an average accuracy of 0.25° to 0.5°, a resolution of 0.01° root mean square, and a micro-saccade resolution of 0.05°. A 9-point calibration was performed for each participant with an average error of ≤0.5°. We no longer used flicker to capture attention to the fixation point at the beginning of the trial as we were using the gaze-contingent display. At the beginning of each trial, a central fixation dot was present and the participant pressed a button on a control pad to begin a trial. To ensure central fixation, we used a “fixation failsafe” algorithm such that, if the participant’s gaze was not within 0.5° of the fixation dot when they pressed the button to initiate the trial, the trial did not proceed. Then, a white dot 0.5° in diameter flashed at the target object’s location for 120 milliseconds as a sudden onset cue. However, in contrast to Experiment 1, this was followed by presentation of the target image for the length of one eye fixation, but with a maximum duration of 1 second. This was accomplished by removing the target image as soon as an eye movement was detected (>30 deg/s peak velocity), or if no eye movement was detected after an image duration of 1 second. Image presentation durations lasting less than 50 milliseconds or greater than 1,000 milliseconds (1 second) were removed from the analysis.

### Results

#### Precursors

With 74 participants participating in 18 trials each, there were a total of 1,332 observations. Prior to any analyses, 3 trials from each participant were filtered to remove 222 trials that contained erroneously generated images.^8^ This resulted in a total of 1,110 observations remaining for the final analyses.

#### Object Identification Accuracy

Results were analyzed similarly to the method of Experiment 1 (see Table 2). As in Experiment 1, the results from Experiment 2 showed that the model with the optimal fit for random effects included by-subject and by-item random intercepts. Furthermore, the model that included the fixed effect of Bouma factor (−441.95) provided a significantly better fit to the data than the null model with the same random-effect structure (−448.61; χ^2^ = 13.38, *df* = 1, *p* < .001). Another important distinction between Experiment 1 and Experiment 2 was that the amount of time available for participants to view the image was controlled by how long they were able to maintain gaze at the center of the display. Specifically, in Experiment 1, the viewing time was fixed by the 80-millisecond target presentation, whereas in Experiment 2, viewing times ranged from 51 to 970 milliseconds. This provided a means of testing whether viewing time (i.e., fixation duration) interacted with Bouma factor as a fixed effect, and whether there was substantial variability in rate of information processing over time across participants as a random effect. However, when the centered fixation duration was included as a fixed effect (−440.72), it provided no improvement to model fitness (χ^2^ = 3.78, *df* = 3, *p* = .43) and therefore had no effect on object recognition accuracy. Thus, the same generalized linear equation as before (i.e., Equation 1) was used to fit the results from Experiment 2.

**Table 2.** Model Fitness Comparisons for Experiment 2.

Fixed effects	By-subject random effects	Model log likelihood (by-subject only)	Additional by-item random effects	Model log likelihood (by-subject + by-item)
Intercept (null model)	Intercept	−480.33 (2)	Intercept	−448.64 (3)
Bouma factor	Intercept	−476.86 (3)	Intercept	−441.95 (4)*
Bouma factor	Bouma factor	−476.86 (5)	Intercept	−441.95 (6)
Bouma Factor × Fixation Duration	Intercept	−475.82 (5)	Intercept	−440.72 (6)
Bouma Factor × Fixation Duration	Bouma factor	−475.82 (7)	Intercept	−440.72 (8)
Bouma Factor × Fixation Duration	Fixation duration	−475.44 (7)	Intercept	−440.06 (8)
Bouma Factor × Fixation Duration	Bouma factor + fixation duration	−475.41 (10)	Intercept	−440.06 (11)
Bouma Factor × Fixation Duration	Bouma Factor × Fixation Duration	−475.33 (14)	Intercept	−439.73 (15)

Columns 1, 2, and 4 detail the contents of the model, whereas Columns 3 and 5 detail model performance in terms of log-likelihood values. Model *df* are in parentheses.Column 1: The *Fixed Effects* structures for the models being compared. These include a null/intercept model on Row 1, which serves as a random chance/baseline comparison. Rows 2 and 3 include the fixed effect of Bouma factor. Rows 4 to 8 include fixed effects of the interaction between Bouma factor and fixation duration.Column 2: The *By-Subject Random Effects* structures include measures of variability in object recognition across subjects. Rows 1, 2, and 4 show *Intercept models*, which account for individual differences for overall object recognition (i.e., random subject intercepts). Rows 3 and 5 show the random slope of Bouma factor, which include models with individual differences in relation to the impact of Bouma factor on object recognition accuracy (i.e., random subject slopes). Row 6 shows the random slope of fixation duration, which includes the individual differences for object recognition accuracy as a function of fixation duration. Row 7 shows the random slope of Bouma factor and fixation duration. Row 8 shows the random slope of the interaction between Bouma factor and fixation duration.Column 3: Log-likelihood values for the *By-Subject Only* random-effect models.Column 4: The *By-Item Random Effects* structures include measures of variability in object recognition across base images.Column 5: Log-likelihood values for the *By-Subject + By-Item* random-effect models.*Denotes the model with the combined largest log likelihood with the smallest *df*. This model included the fixed effect of Bouma factor, the *By-Subject Random Effect* of Intercept, and the *By-Item Random Effect* of Intercept of base image.

**Figure 6.**
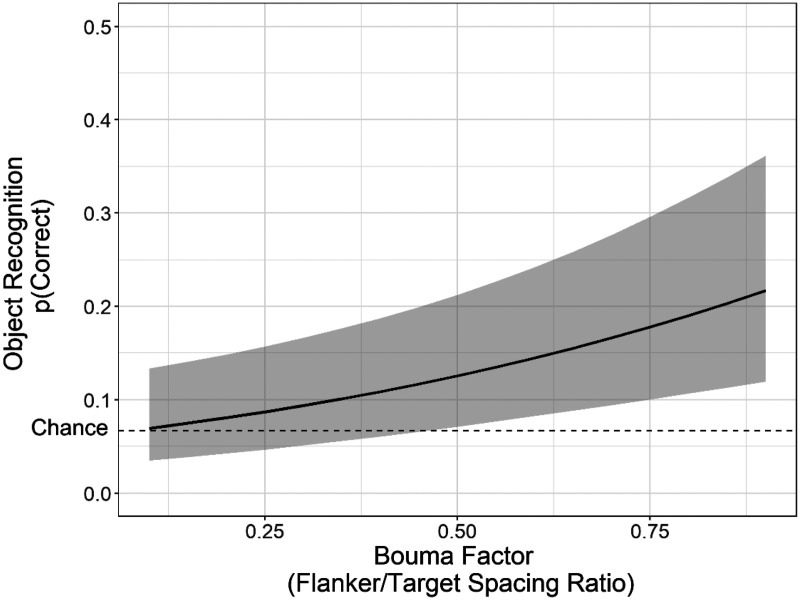
Fitted mean accuracy for object identification as a function of the fixed effect of Bouma factor, based on the best-fit mixed-logit model from Experiment 2. The log-odds for object recognition have been transformed with an inverse-logit function to provide proportion correct for the logistic function. The range of Bouma factor extends from the minimum (0.18) to the maximum (0.81) values used in this study (as illustrated in Figure 2). Error bars represent a 95% confidence interval above and below the mean (using model parameter estimates obtained from lme4). The dotted line indicates the chance level of accuracy (6.7%).

Mean accuracy was approximately 11.8% (α = −2.01, *z* = −6.25, *p* < .001), and slightly above the chance-level accuracy of 6.67%. As shown in Figure 6, target identification accuracy increased significantly with Bouma factor (β = 1.64, *z* = 3.69, *p* < .001). This replicates the primary results of Experiment 1 of crowding of objects in real-world scenes and shows that those results cannot be explained by subjects fixating the target object. The fact that object recognition improved as flanker spacing increased provides evidence for the general phenomenon of crowding. However, recognition accuracy as a function of Bouma factor seemed to improve at a more gradual rate than in Experiment 1.

**Figure 7.**
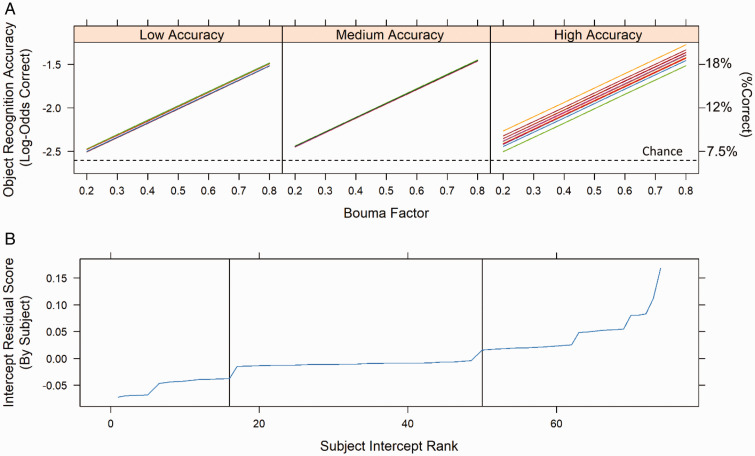
Fitted log-odds accuracy as a function of Bouma factor for individual participants in Experiment 2. A: Proportion correct is indicated on the right y-axis. Each line represents an individual participant’s fitted accuracy for the random slopes model, while the panels are divided among participants with low (left), medium (center), or high (right) levels of mean accuracy for object recognition accuracy. This is not the model used to generate Figure 6 because it had additional flexibility for individual subjects’ slopes but does demonstrate that the addition of random slopes provided no benefit for improved goodness of fit. Notice that the slope for object recognition accuracy across Bouma factor does not differ as a function of overall accuracy (i.e., intercept) even though slopes were able to vary. Group divisions were based on random-effect residual scores (as shown in Figure 5B). The horizontal dotted line indicates the centered model intercept. B: Mean accuracy (intercept) residual scores for individual subjects. Individual subjects are ordered by rank, and the vertical lines indicate divisions used to generate the accuracy groups in Figure 7A.

Similar to Experiment 1, Experiment 2’s model comparisons found that the optimal random-effect structure was a random-intercept model. This model was tested against a model with a random slope of Bouma factor, which fitted individual subjects’ slopes for accuracy as a function of Bouma factor (see models in Line 3 of Table 2). However, this additional level of model complexity provided identical goodness of fit, and thus the simpler random-intercept model was found to be optimal. Again, this result demonstrates that there were minimal individual differences across participants with respect to the effect of Bouma factor (i.e., slope) on overall recognition accuracy (i.e., intercept, see Figure 7A). This is further supported by the fact that the fixed-effect correlation between subject intercept and Bouma factor slope was not significant, *r*(74) =.08, *p* = .51.

Figure 7 also illustrates some characteristics of the results in Experiment 2. The reason that Figure 7A appears to contain only a single line for the Medium Accuracy tertile is that there was only a single accuracy level in that tertile. This is indicated in Figure 7B, which shows that the residual score for the Medium Accuracy tertile had only a single value. Similarly, in Figure 7A, there are only two slopes for the Low Accuracy tertile, which is because there were only two scores in that tertile, as shown in Figure 7B. In fact, in the Medium Accuracy tertile, the single score was 2/15, or 13.3% correct, and in the Low Accuracy tertile, the two scores were 1/15 (6.7% correct), and 0/15 (0% correct). The Medium and Low Accuracy tertiles therefore had a floor effect, producing scant information about the relationship between Bouma factor and accuracy. On the other hand, there was much more variability in the High Accuracy tertile, as shown by the multiple different (but highly similar) slopes in Figure 7A, and multiple different residuals in Figure 7B. The High Accuracy tertile provided the variability in accuracy as a function of Bouma factor which allowed the model to determine the slopes for that function. The model was then able to extrapolate that function to the Medium and Low Accuracy tertiles, providing one and two slopes for the Medium and Low Accuracy tertiles respectively. Despite this, further analysis has shown that the three accuracy tertiles demonstrate the same general pattern: more incorrect responses as Bouma factor increased. It is for this reason that the slopes for accuracy as a function of Bouma factor were roughly equivalent across the accuracy tertiles.

## General Discussion

Experiment 2 replicated the crowding effect found in Experiment 1. Thus, we conclude that crowding can indeed occur within the context of naturalistic scenes in which both the target objects and their flankers are consistent with those scenes. Thus, unlike in Wijntjes and Rosenholtz (2018), the context of the surrounding consistent scene information was not enough to overcome the crowding produced by flanking objects. A likely reason for the contrasting results between studies is that this study systematically varied the spacing between flanker and target objects at a range around Bouma’s constant, such that images would be more or less likely to produce the crowding effect. This was not the case in the study by Wijntjes and Rosenholtz (2018), where an aperture was placed around objects in real-world scenes to either include or exclude nearby scene information. The fact that we created different versions of the same images in which only the target flanker spacing varied provides additional evidence that scene context was not sufficient to counteract the crowding effect. Instead, this study provides more rigorous and controlled experimental support for the earlier demonstration of crowding of a single object in a single scene by Whitney and Levi (2011) and for the possible crowding effects in street scenes shown by Sanocki et al. (2015). Furthermore, this study did so with a relatively large number of naïve participants while carefully controlling target and flanker retinal eccentricities by using a gaze-contingent fixation fail-safe algorithm.

Although object recognition accuracy was somewhat low in this study, most participants were well above chance-level accuracy despite using their peripheral vision to recognize objects. Both experiments found the same general pattern of crowding in real-world scenes, but performance was better overall in Experiment 1 than in Experiment 2. One explanation for the better performance in Experiment 1, which did not use eye tracking to ensure central fixation, is that participants adopted the strategy of fixating off center in order to view the target objects in central vision. However, given that participants did not know the locations of the targets prior to each trial, the interstimulus interval between the location prime and the onset of the image was too brief to make an eye movement. Adopting such a strategy is just as likely to have impaired performance as it was to have improved it. Alternatively, in Experiment 2, the target image disappeared from the screen as soon as participants moved their eyes from fixation, and therefore untrained observers may have had difficulty in maintaining stable central fixation while they covertly attended to the peripheral target. The median viewing time for images in Experiment 2 was 373 milliseconds, which is slightly longer than the average fixation duration in scenes (330 milliseconds; Rayner, 1998), and was nearly double the sum of the cuing duration and target duration in Experiment 1 (120 milliseconds + 80 milliseconds, respectively, = 200 milliseconds). Although trained observers who are able to maintain longer fixations (e.g., 1,000 milliseconds) while attending to peripheral targets might perform better than the untrained observers in Experiment 2, the current results with untrained observers likely represent the difficulties caused by crowding in normal real-world scene perception. This result is consistent with results from Wallace et al. (2013), who showed that identification of crowded letters in the periphery did not benefit from processing times beyond 250 milliseconds.

Conversely, data from a visual search task in which participants were required to maintain gaze at a central location showed that detection rates benefitted from fixation durations lasting up to 3 seconds, and that detecting targets positioned 12° from the fovea occurred after approximately 400 to 1,200 milliseconds of search time (Motter & Simoni, 2008). The results from Experiment 2 in this study clearly demonstrate that accounting for viewing time did not provide any benefit to object recognition accuracy, which suggests that the crowding phenomenon may hinge entirely on *signal* data limitations, rather than processing resources, like processing time (Norman & Bobrow, 1975). This is not to say that crowding is completely independent of other processing resources, like attention. For instance, Yeshurun and Rashal (2010) have previously reported that the critical spacing value for crowding in peripheral vision (i.e., the minimal spacing at which crowding no longer occurs) can be decreased using transient attentional cues. Consistent with our results, Yeshurun and Rashal also found no change in accuracy with increased viewing time. Recent computational models of crowding have found evidence that the visual system’s sparse coding for early gist information is quite accurate at predicting the crowding effect across multiple levels of processing (Chaney et al., 2014). Given that gist is rapidly acquired and heavily dependent upon peripheral vision (Larson & Loschky, 2009; Wang & Cottrell, 2017), it is unsurprising that we did not find an effect of viewing time.

The data presented in this study do show that crowding can occur in real-world scenes, even when the target and flanker objects are semantically consistent with the scene. However, the precise mechanisms which contribute to crowding are still being investigated. Specifically, whether crowding is a product of spatial pooling (Dakin et al., 2010; Greenwood et al., 2010; Parkes et al., 2001; Wallis & Bex, 2012; Wolford, 1975), localization errors (Freeman et al., 2011; Huckauf & Heller, 2002; Poder & Wagemans, 2007; Strasburger, 2014; Strasburger & Malania, 2013), or both (Hanus & Vul, 2013; Strasburger, 2005; Strasburger et al., 1991), is an ongoing debate. For instance, Wallis and Bex (2012) found compelling evidence that crowding occurs in the visual stream prior to object recognition. In that study, participants were required to detect textured, elliptical patches—called *dead leaves*—in natural scenes. They found that the degree to which participants could detect artifacts in natural scenes at different eccentricities depended on the size of the patches as well as the local contrast of the image structure surrounding. More importantly, they found that the relationship between eccentricity, local root mean square contrast, and patch size closely resembles receptive field sizes in V1, which would suggest that crowding can occur before object recognition. Nevertheless, there is a wealth of evidence demonstrating that crowding can occur at later stages of processing (e.g., V4; van den Berg et al., 2010) when object features are preserved but are not effectively isolated from the features of nearby objects (Pelli et al., 2004). Therefore, an analysis of errors in Experiments 1 and 2 may provide further insights into nature of the crowding effect in natural scenes.

We carried out an exploratory analysis of the errors to see whether there was evidence of localization errors. We found that approximately 41% and 44% of all errors, in Experiments 1 and 2, respectively, were due to mistaking flanker objects for the targets. Note that those percentages are considerably higher than the chance probability of randomly selecting one of the four flankers from among the 14 distractor options (≈ 28.5%). This result is consistent with numerous prior studies (Chung et al., 2003; Estes et al., 1976; Huckauf & Heller, 2002; Strasburger, 2005; Strasburger et al., 1991; Strasburger & Malania, 2013; Vul et al., 2009; Wolford & Shum, 1980; see Strasburger, 2020, for review). For example, Strasburger (2005, p. 1028) found up to 52% of errors were localization errors in crowding. However, the Bouma factor did not appear to affect the likelihood of mistakenly selecting a flanker object in either Experiment 1 (β = −0.634, *z* = −1.37, *p* = .17) or Experiment 2 (β = −0.078, *z* = −0.16, *p* = .87). Nevertheless, the general finding that participants erred by confusing nearby objects with target objects is partially in agreement with the crowding literature.

As noted earlier, Wijntjes and Rosenholtz (2018) raised the important question regarding the degree to which scene context provides protection from crowding. In their Experiment 1, they estimated crowding in scenes by increasing the size of an aperture around a target object, with the assumption that more of the viewable scene would include more crowding objects. Because accuracy improved with increased aperture size, they concluded that scene information provided a protective effect against crowding. Although they found that more flanker objects were generally visible with a larger aperture, the number of objects within the critical (crowding) radius was not included as a fixed parameter in their model. In contrast to Wijntjes and Rosenholtz, the models evaluated in our experiments explicitly tested whether object/flanker distance contributed to a meaningful difference in object recognition accuracy and found that it did. Our results do not rule out the hypothesis that natural scenes provide some protection against crowding, as even the smallest target/flanker ratios resulted in above-chance recognition accuracy. However, the design of our study also manipulated the Bouma factor of targets and flanker objects across different versions of the same scene, effectively holding scene context constant. The comparisons made in our models indicated that target/flanker distance contributes a meaningful, generalizable effect (Baayen et al., 2008) on object recognition in simulated natural scenes, and that this crowding effect is similar to the findings of other less naturalistic crowding experiments (Levi, 2008; Parkes et al., 2001; Poder & Wagemans, 2007; Whitney & Levi, 2011).

One limitation of this study is the stimulus set used. On the one hand, by using interior design software to create the stimuli in this study, we had the advantage of being able to control target/flanker distances while creating highly realistic scene stimuli in which the objects were semantically consistent with the scenes. On the other hand, the task of creating such stimuli was far from trivial, thus limiting our total number of scenes, and the range of scene categories, as compared with the studies in Wijntjes and Rosenholtz (2018), who tested for crowding in a wider variety of natural scene images. Future research will need to go beyond the assumption *that* individual differences in participants and scene context affect crowding and determine *how* these individual differences affect crowding.

One consistent result across participants was that object recognition was quite low across most participants, even when the flanker objects were far from the targets, and likely not crowded. Low recognition accuracy could be attributed to the large distance of the target objects from the center of gaze (11.3°–13.8° eccentricity) as well as the added difficulty of a 15-alternative forced choice recognition task. As reported in Experiment 2, the Bouma factor that yielded above-chance object recognition was approximately 0.5. Bouma (1970) originally found that the critical value for crowding was approximately 0.5, and many other studies have found similar critical values (Pelli, Tillman, et al., 2007; Strasburger, 2020; Strasburger et al., 1991). However, the interpretation of the critical value derived from our study differs from these prior studies because they reflect disparate points on the psychometric function. Specifically, the critical value from our study represents the point at which object recognition is minimally above chance, whereas the original Bouma factor reflects the point at which crowding *diminishes*. To frame our results in terms of the broader context of the crowding literature, it is important to consider several components of our task and stimuli and their impact on performance.

First and foremost, object identification was fairly difficult, regardless of the Bouma factor, with mean accuracy failing to exceed 40% in Experiment 1 and 22% in Experiment 2 (with chance being 1/15 = 6.6%). Although the slope for accuracy as a function of Bouma factor was positive, recognition accuracy did not seem to reach asymptotic performance, regardless of the spacing between flankers and targets. Extrapolating perceptual performance from the available data suggests that the Bouma factor necessary to reach 80% accuracy would be between 1.55 (Experiment 1) and 2.55 (Experiment 2), both substantially larger than those estimated by (Pelli, Tillman, et al., 2007). Indeed, given that our target eccentricities were roughly 10°, Bouma factors of 1.5-2.5 would require flankers at 15° to 25° from the target, many of which would be outside of the visible image space. This level of added task difficulty made estimating the entire psychometric function for accuracy as a function of Bouma factor impractical, if not impossible. Therefore, future investigations of crowding in natural scenes should explore peripheral object recognition accuracy over a wider range of target eccentricities and target sizes, contextual coherence, and with varying numbers (and arrangements) of flankers to account for the whole range of the psychometric function.

It is possible that multiple factors affect crowding of objects in natural scenes, as proposed by Wijntjes and Rosenholtz (2018). For instance, if the semantic context of the scene is capable of counteracting the crowding effect, then the critical spacing for targets and flankers should decrease when targets and objects are consistent with scene category and increase when they are inconsistent with their scene category (Wijntjes & Rosenholtz, 2018). Conversely, it may be the case that crowding is unaffected by semantic consistency versus inconsistency but is dependent upon abstract representations of scene space (e.g., openness, navigability, depth; Greene & Oliva, 2009). Such a distinction may be difficult to tease apart, given that high- and low-level representations of scenes are strongly correlated with each other (Groen et al., 2017). However, distinguishing between categorical and spatial influences of objects in natural scenes will be crucial to forming a more complete model of crowding in natural scenes.

In sum, this study has shown clear evidence of crowding of objects embedded within realistic real-world scenes, in the general sense of the phenomenon. Furthermore, this study showed crowding of objects in realistic scenes while carefully controlling for both the retinal eccentricity of targets and the spacing between targets and flankers according to the Bouma factor. Additional control over target and flanker distance was enforced with the use of gaze-contingent displays. Further such studies are called for to investigate the range of critically important questions regarding crowding (Pelli & Tillman, 2008; Strasburger, 2020; Strasburger et al., 2011; Whitney & Levi, 2011) within the context of real world scenes (e.g., simulated driving). Such studies will play an important role in understanding peripheral vision in scene perception.

## Notes

1 It is important to note that the vast literature on crowding over the last two decades has shown a large number of other variables can influence the critical spacing necessary to produce the crowding phenomenon (Whitney & Levi, 2011). However, discussion of most of these factors is beyond the scope of this study.

2 Natural images have also been manipulated using algorithms to create pseudo-crowding (Freeman & Simoncelli, 2011).

3 Thanks to Michael R. Luczak for the design and creation of the stimuli and to Kevin K. Rooney for assistance in stimulus design.

4 The conventional usage of the term *Bouma factor* (or *Bouma’s factor*) is as the parameter in Bouma’s law, which describes the ratio between critical flanker spacing and target eccentricity, where critical spacing refers to the spacing necessary to achieve a specific criterion level of accuracy (Pelli, Tillman, et al., 2007; Strasburger, 2020). Because our study manipulated target eccentricity and flanker spacing through the method of constant stimuli and not an adaptive threshold, here we use the Bouma factor in an image as a variable to quantify the range of target-flanker spacing as a value that is normalized across target eccentricity in each image.

5 Below chance (i.e., <6.7%) accuracy was found for 13 of the 44 participants. However, comparisons of the results with and without the below-chance participants did not substantially affect the slope for Bouma factor. Therefore, the reported results for Experiment 1 were generated using data from all participants.

6 The reported comparison was between two fixed-effect models that had by-subject random-intercepts and by-item random effects.

7 Prior to analysis, Bouma factor was centered by the mean Bouma factor for all images, or 0.45. Therefore, the intercept (α) should be interpreted as accuracy of the mean Bouma factor of images used in the study.

8 Twenty-three participants demonstrated accuracy below chance (6.7%) accuracy. However, a comparison between results with and without the low performing individuals showed no meaningful difference. Therefore, results reported include all participants.
